# UCP2 modulates cardiomyocyte cell cycle activity, acetyl-CoA, and histone acetylation in response to moderate hypoxia

**DOI:** 10.1172/jci.insight.155475

**Published:** 2022-08-08

**Authors:** Vagner O.C. Rigaud, Clare Zarka, Justin Kurian, Daria Harlamova, Andrea Elia, Nicole Kasatkin, Jaslyn Johnson, Michael Behanan, Lindsay Kraus, Hannah Pepper, Nathaniel W. Snyder, Sadia Mohsin, Steven R. Houser, Mohsin Khan

**Affiliations:** 1 Center for Metabolic Disease Research (CMDR),; 2 Cardiovascular Research Institute (CVRC), and; 3 Department of Cardiovascular Sciences, LKSOM, Temple University, Philadelphia, Pennsylvania, USA.

**Keywords:** Cardiology, Metabolism, Cell cycle, Hypoxia, Uncoupling proteins

## Abstract

Developmental cardiac tissue is regenerative while operating under low oxygen. After birth, ambient oxygen is associated with cardiomyocyte cell cycle exit and regeneration. Likewise, cardiac metabolism undergoes a shift with cardiac maturation. Whether there are common regulators of cardiomyocyte cell cycle linking metabolism to oxygen tension remains unknown. The objective of the study is to determine whether mitochondrial UCP2 is a metabolic oxygen sensor regulating cardiomyocyte cell cycle. Neonatal rat ventricular myocytes (NRVMs) under moderate hypoxia showed increased cell cycle activity and UCP2 expression. NRVMs exhibited a metabolic shift toward glycolysis, reducing citrate synthase, mtDNA, mitochondrial membrane potential (ΔΨm), and DNA damage/oxidative stress, while loss of UCP2 reversed this phenotype. Next, WT and mice from a global UCP2-KO mouse line (UCP2KO) kept under hypoxia for 4 weeks showed significant decline in cardiac function that was more pronounced in UCP2KO animals. Cardiomyocyte cell cycle activity was reduced, while fibrosis and DNA damage was significantly increased in UCP2KO animals compared with WT under hypoxia. Mechanistically, UCP2 increased acetyl-CoA levels and histone acetylation, and it altered chromatin modifiers linking metabolism to cardiomyocyte cell cycle under hypoxia. Here, we show a potentially novel role for mitochondrial UCP2 as an oxygen sensor regulating cardiomyocyte cell cycle activity, acetyl-CoA levels, and histone acetylation in response to moderate hypoxia.

## Graphical abstract

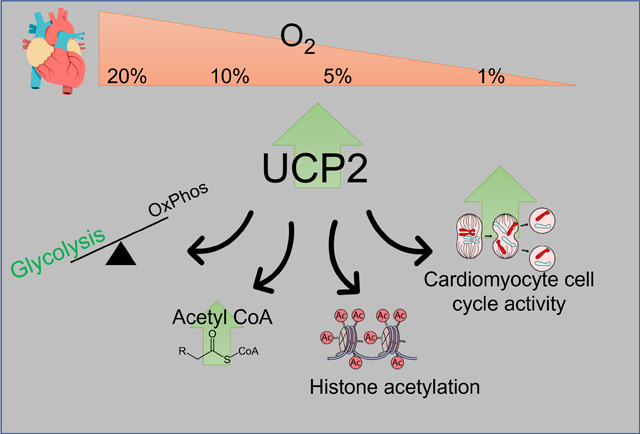

## Introduction

The adult human heart possesses limited cardiomyocyte (CM) turnover and is unable to respond to the massive cell loss that underlies cardiac injury (1). In contrast, the mammalian developing heart is a proliferative organ consisting of rapidly dividing CM (2, 3). Recent studies implicate unique CM transcriptional and functional profile linked to enhanced proliferation rates during development (4). Additionally, changes in cardiac environment such as nutrient availability and oxygen tension support CM proliferation (5, 6). Immediately after birth, exposure to oxygen-rich ambient environment has been identified as one of the earliest events driving CM cell cycle arrest (7). In the adult heart, a pool of hypoxic CM has been found as responsive to proliferative signals (8) further highlighting the role of oxygen tension within the heart as a mediator of CM proliferation and cell cycle. Although several reports have suggested that oxygen metabolism and oxidative stress play a pivotal role in regulating the proliferative capacity of CM (3, 7, 8), how CM adapt to low-oxygen levels and the molecular signaling underlying hypoxia-induced CM proliferation remain largely unknown.

Cardiac metabolism has been recently linked to regulation of CM cell cycle activity (9–11). Developmental cardiac tissue operates under glycolytic metabolism that shifts to oxidative phosphorylation after birth in response to increasing oxygen tension and is associated with loss of cardiac regenerative properties (7). Adult CM exposed to hypoxia exhibit glycolytic metabolism and increased cell cycle activity (8, 12, 13). Nevertheless, whether there are common regulators of CM cell cycle activity that link metabolism and oxygen tension remains undefined. Uncoupling proteins are a family of anion transporters present in the inner mitochondrial membrane and are closely related to changes in energy metabolism by preventing mitochondrial glucose oxidation and facilitating glycolysis via substrate shunting mechanism (14–16). We have recently identified mitochondrial uncoupling protein 2 (UCP2) to be highly expressed in the neonatal heart and crucial for maintenance of the glycolytic metabolism, proliferation, and survival of neonatal cardiac cells (17). Aging of the cardiac tissue is associated with loss of UCP2 together with induction of oxidative phosphorylation in cardiac cells (17). Thus, we hypothesized that UCP2 may act as an early adaptative response protein for sensing changes in oxygen tension regulating metabolism and CM cell cycle activity in the heart.

Here we show that UCP2 is sensitive to oxygen levels being highly expressed in response to moderate hypoxia in the heart. Using in vitro and in vivo approaches, we demonstrate that UCP2 is required for both metabolic reprogramming toward glycolysis and CM cell cycle activity under hypoxic conditions preventing DNA damage and regulating epigenetic landscape. In addition, UCP2 was shown to increase cytosolic acetyl-CoA generation and histone acetylation possibly affecting the expression of CM cell cycle genes, thereby linking metabolism with regulation of CM cell cycle under moderate hypoxia.

## Results

### Moderate hypoxia promotes UCP2 expression and CM cell cycle activity.

**Figure 1:**
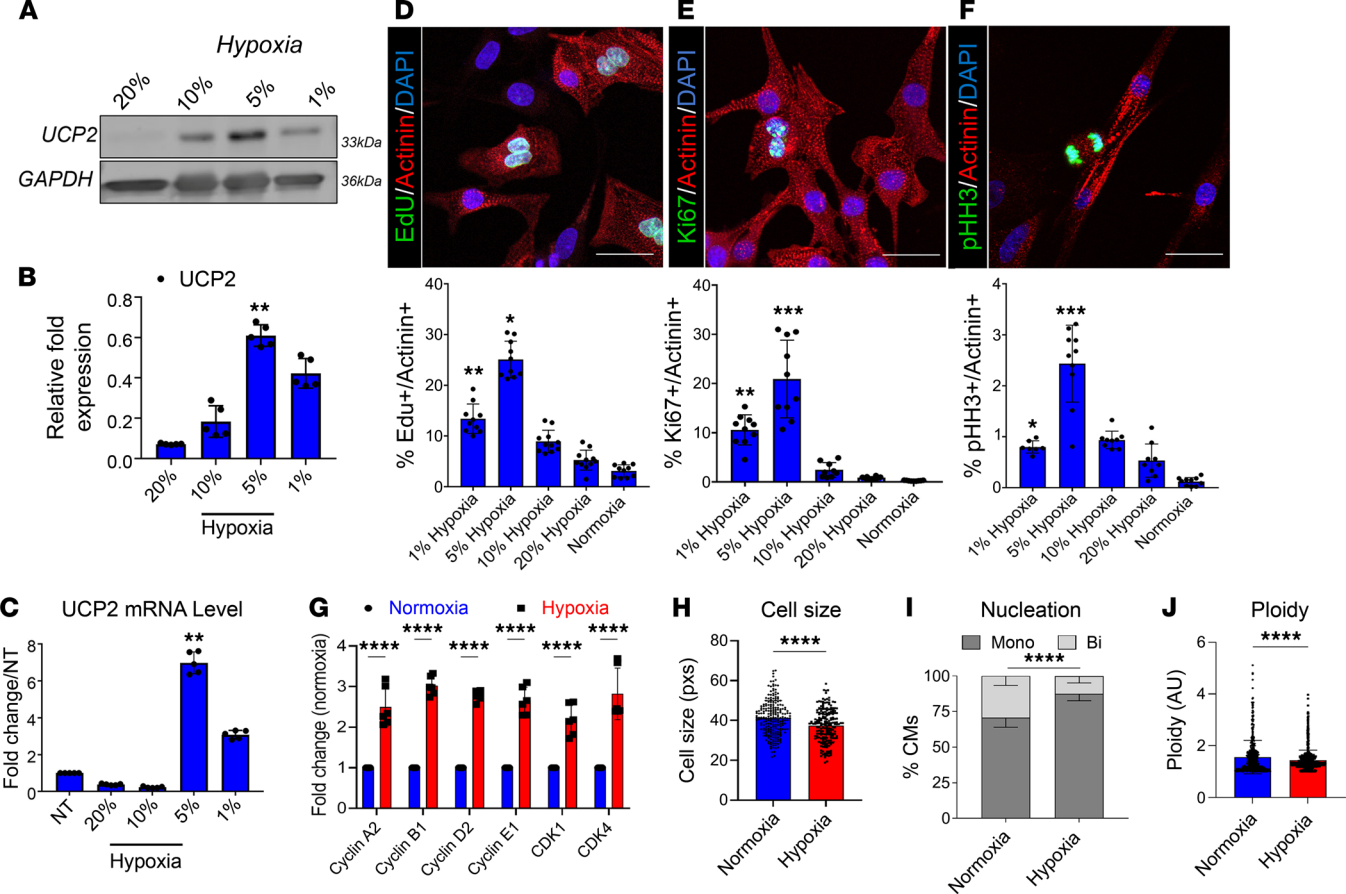
Moderate hypoxia drives UCP2 expression associated with increased cell cycle activity in neonatal cardiomyocytes. (**A**–**C**) UCP2 expression progressively increases with hypoxia in neonatal rat ventricular myocytes (NRVMs) as measured by Western blot (**A** and **B**) and qPCR (**C**). Percentages represent oxygen levels (*n* = 5). (**D**–**F**) NRVM cell cycle activity is significantly enhanced after conditioning in moderate hypoxic (5% oxygen) environment, as evidenced by increased levels of EdU incorporation (**D**), and Ki67 (**E**), and phosphorylated Histone H3 levels (**F**). EdU, Ki67, and pHH3, green; α sarcomeric actinin, red; nuclei, blue. Scale bar: 40 μm (*n* = 5). (**G**) mRNA levels of cell cycle markers are increased in moderate hypoxic NRVMs. (*n* = 6). (**H**–**J**) Cellular morphology is altered in moderate hypoxia featuring smaller cell size (**H**), fewer nuclei per cell (**I**), and reduced DNA content (**J**) (*n* = 3). Normoxia represents regular atmospheric oxygen levels (~21%). Normoxia versus Hypoxia *P < 0.05, **P < 0.01, ***P < 0.001, ****P < 0.0001. Data from **A**–**G** were analyzed using Kruskal-Wallis test with Dunn’s correction for multiple comparisons; for **H**–**J**, unpaired 2-tailed Student’s *t* test was applied.

Our previous work identified UCP2 to be highly expressed in the neonatal heart and lost upon cardiac aging (17). As the neonatal heart operates under low oxygen tension (7), we hypothesized, UCP2 may be responsive to changes in oxygen tension. We cultured neonatal rat ventricular myocytes (NRVMs) immediately after isolation under 21% oxygen that was reduced every 24 hours to 10%, 5%, and 1% (Supplemental Figure 1A; supplemental material available online with this article; https://doi.org/10.1172/jci.insight.155475DS1). Subsequent molecular evaluation revealed increased UCP2 expression levels in response to moderate hypoxia (5%) at both mRNA and protein levels with a decrease associated with severe hypoxia (1%) (Figure 1, A–C). Since UCP2 expression is downregulated in adult hearts (17), we sought whether adult CM could upregulate UCP2 in low-oxygen conditions. Immunocytochemistry analysis of adult feline CM revealed enhanced UCP2 levels when cultured under moderate hypoxia (5%) (Supplemental Figure 1B). The next consideration became whether increased levels of UCP2 in response to hypoxia impact CM cell cycle activity. For this purpose, NRVMs cultured under hypoxic conditions as described above were treated with thymidine nucleoside analog EdU for a pulse-chase experiment. As a result, EdU labeling was significantly higher in NRVMs exposed to moderate hypoxia (5%) (Figure 1D) compared with other oxygen concentrations and normoxic NRVMs, indicating enhanced DNA synthesis. Interestingly, reducing oxygen tension further to 1% decreased EdU^+^ NRVMs, suggesting severe hypoxia to limit CM cell cycle activity. Similarly, increased cell cycle markers Ki67^+^ and pHH3^+^ cells were observed in moderate hypoxia condition when compared with other oxygen concentrations and normoxia (Figure 1, E and F). Furthermore, mRNA levels of cell cycle checkpoint markers were also enriched in NRVMs under moderate hypoxia compared with normoxia (Figure 1G). Finally, a reduction in cell size (Figure 1H) along with an increase in mononucleated diploid CM (Figure 1I) and reduction in ploidy (Figure 1J) were also observed in NRVMs under moderate hypoxia compared with normoxia. Taken together, these results suggest that UCP2 expression is sensitive to oxygen levels and that its expression is enhanced in response to hypoxia in both neonatal and adult CM, associated with enhanced CM cell cycle activity.

### Hypoxia-induced UCP2 upregulation is associated with alteration of CM metabolism and reduction in DNA damage.

**Figure 2:**
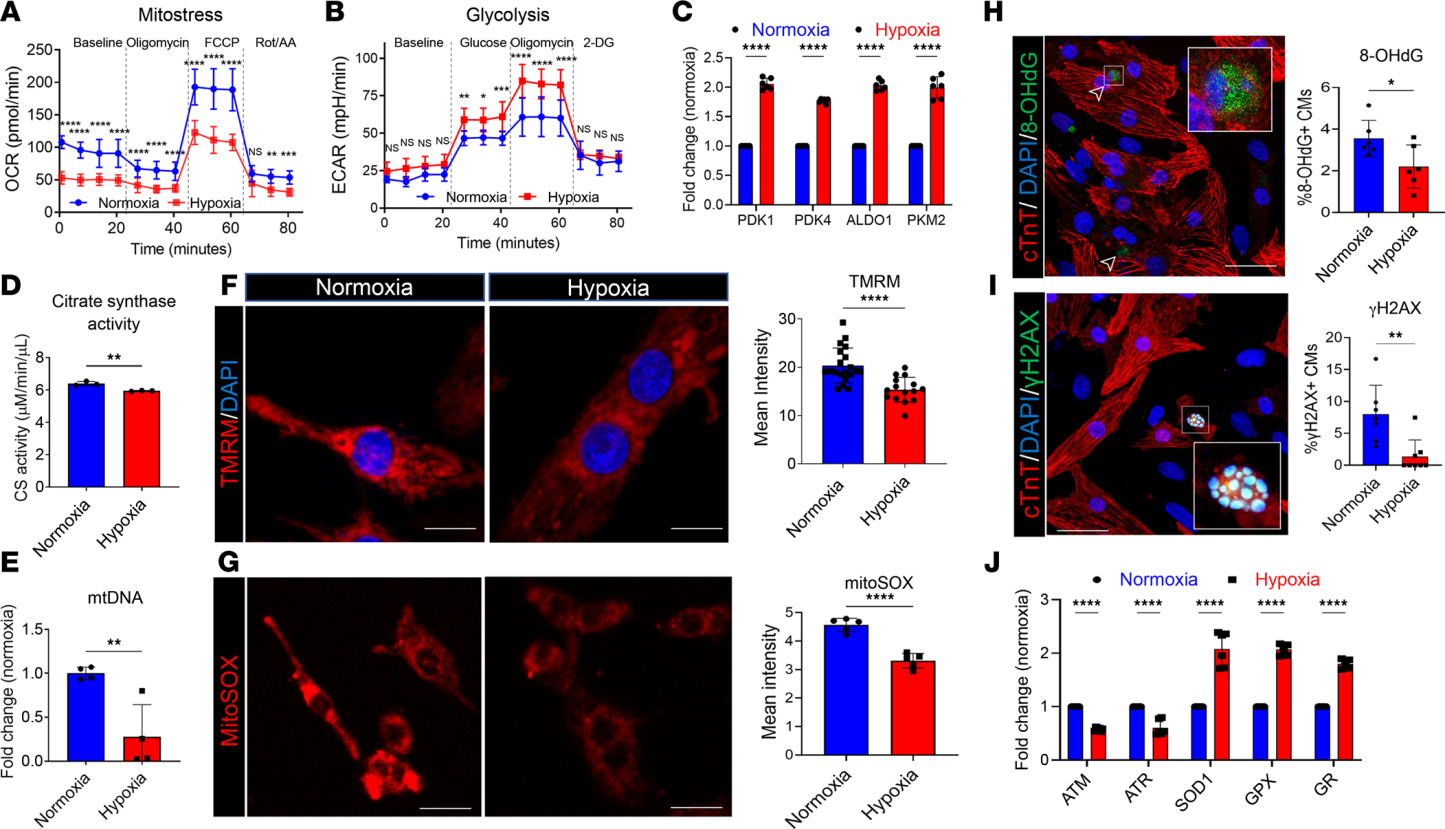
Hypoxia-induced UCP2 overexpression is associated with glycolytic metabolism and reduced oxidative damage in cardiomyocytes. (**A** and **B**) Measurement of oxygen consumption rate (OCR) (**A**) and extracellular acidification rates (ECAR) (**B**) by Seahorse Bioanalyzer show decreased mitochondrial respiration and increased glycolysis, respectively, in NRVMs after 48 hours under hypoxia compared with 48 hours under normoxic conditions (*n* = 12 replicates/condition/3 independent experiments). Data for OCR/ECAR was normalized to cell number. (**C**–**E**) Further validation shows increased mRNA levels of glycolytic enzymes (**C**), reduced citrate synthase activity (**D**), and reduced mitochondrial DNA content (**E**) in moderate hypoxic NRVMs compared with their normoxic counterparts. (*n* = 3/condition/experiment). (**F**) Hypoxic NRVMs demonstrate lower mitochondrial membrane potential visualized by TMRM staining. TMRM, red; nuclei, blue. (*n* = 3). Scale bar: 20 μm. (**G**) Reduced mitochondrial superoxidase production in NRVMs under hypoxia compared with NVRMs in normoxia measured by MitoSOX intensity. Scale bar: 20 μm (*n* = 3). (**H** and **I**) Reduced levels of the oxidative DNA damage marker 8-OHdG (**H**) and DNA double-strand breaks marker γH2A.X (**I**) in NRVMs under hypoxia. 8-OHdG/γH2A.X, green; cardiac troponin T, red; nuclei, blue. Scale bar: 40 μm. (*n* = 3). (**J**) Reduced mRNA expression of DNA damage markers ATM and ATR and increased antioxidant markers SOD1, GPX, and GR in NRVMs under hypoxia compared with normoxia (*n* = 4). *P < 0.05, **P < 0.01, ****P < 0.0001. Data from **A**–**C** and **J** were analyzed using Kruskal-Wallis test with Dunn’s correction for multiple comparisons; data for **D**–**I** were analyzed by Mann-Whitney *U* test.

The developmental heart consists of proliferative CM that operate under glycolytic metabolism (7, 11). After birth, an increase in oxygen levels shifts metabolism toward a more oxidative state that is associated with CM cell cycle arrest (7). To understand how hypoxia induced UCP2 overexpression affects CM bioenergetics, oxygen consumption rate (OCR) and extracellular acidification rate (ECAR), indicators of mitochondrial respiration and glycolytic rate, respectively, were measured using a Seahorse Bioscience XF Analyzer. Results demonstrate that OCR was significantly lower in NRVMs under moderate hypoxia (5%) compared with control cells under normoxia (Figure 2A). In contrast, decreased OCR was compensated with an elevated glycolytic rate in hypoxic myocytes, as shown in Figure 2B, together with increased mRNA expression of glycolytic enzymes (Figure 2C). Further validation showed decreased citrate synthase activity (Figure 2D) and mitochondrial DNA content (Figure 2E) in NRVMs under moderate hypoxia compared with normoxic cells. Moreover, a loss of mitochondrial membrane potential (ΔΨm) was also observed by a reduction in TMRM accumulation (Figure 2F). These results suggest that mitochondrial respiration is decreased, whereas glycolysis is enhanced in CM under moderate hypoxia (5%). As oxidative metabolism is associated with increased production of reactive oxidative species (ROS), we investigated whether ROS production and subsequent DNA damage were altered by hypoxia-induced UCP2 overexpression. Since mitochondria are the major intracellular source of ROS, we first assessed mitochondrial superoxide production by MitoSOX live staining. As expected, NRVMs under moderate hypoxia showed reduced mitochondrial ROS generation (Figure 2G). These findings were further confirmed by immunocytochemistry analyses showing decreased levels of oxidative DNA damage markers 8-OHdG and γ-H2A.X (Figure 2, H and I, respectively) and apoptosis as measured by TUNEL assay (Supplemental Figure 2A), along with decreased mRNA expression of DNA damage–sensing markers such as ATM and ATR and increased expression of antioxidant genes SOD1, GPX, and GR (Figure 2J). Taken together, UCP2 upregulation under moderate hypoxic conditions may trigger a switch from mitochondrial respiration to glycolysis that protects CM from oxidative DNA damage and subsequent apoptosis.

### UCP2 is essential for CM cell cycle activity and metabolic reprogramming under moderate hypoxia.

**Figure 3:**
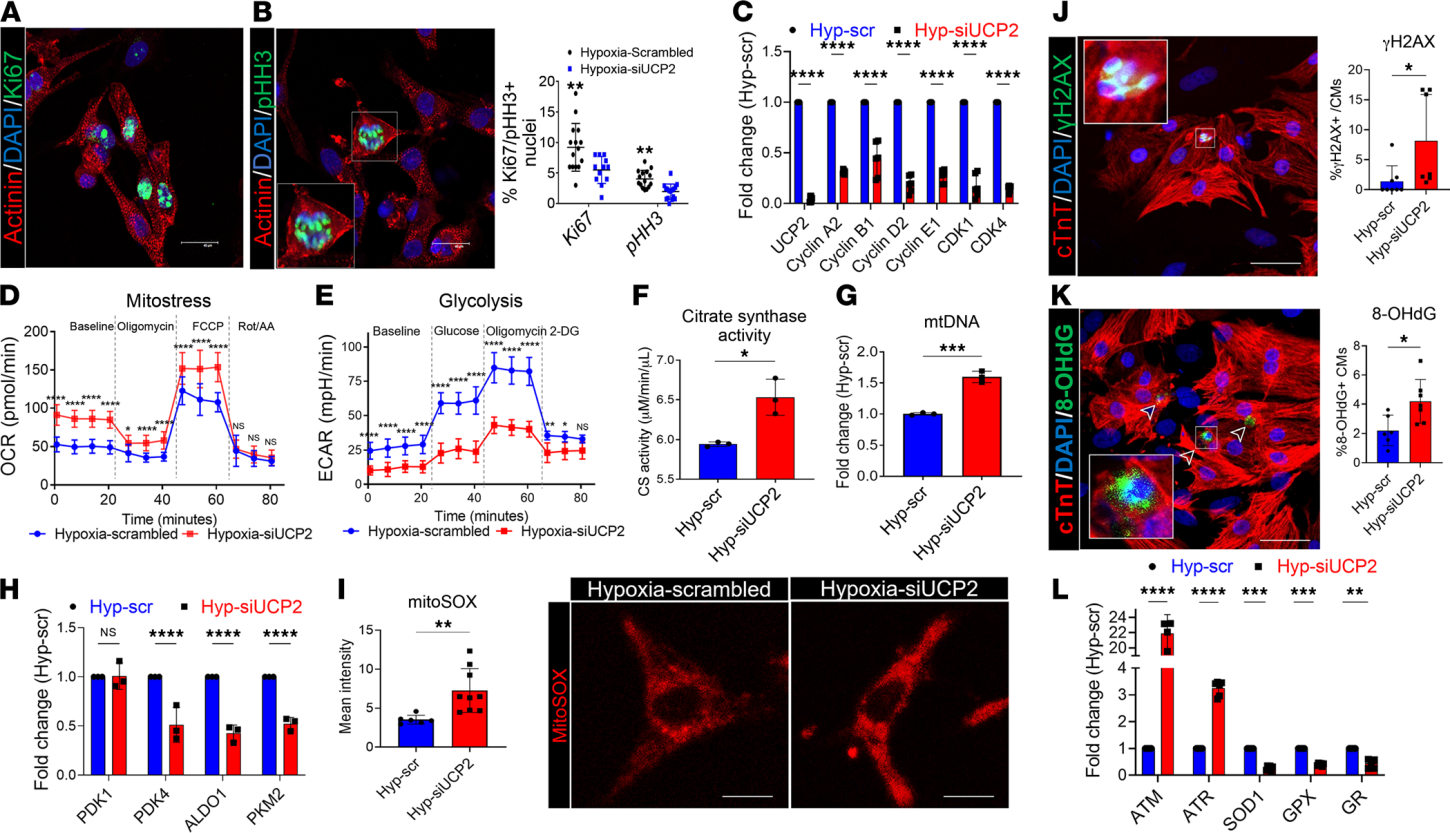
Loss of UCP2 attenuates glycolysis and cell cycle activity, increasing DNA damage in cardiomyocytes under hypoxia. (**A** and **B**) Reduced cell cycle activity in NRVMs under hypoxia treated with 25 nM siUCP2 for 48 hours compared with scrambled siRNA controls and measured by immunostaining for the proliferation markers Ki67 and pHH3. Ki67/pHH3, green; α sarcomeric actinin, red; nuclei, blue. Scale bar: 40 μm (*n* = 3). (**C**) mRNA expression levels validating the efficiency of siRNA-mediated silencing of UCP2 and reduced mRNA levels of proliferation marker genes. (**D** and **E**) Measurement of oxygen consumption rate (OCR) and extracellular acidification rates (ECAR) by Seahorse Bbioanalyzer show increased mitochondrial respiration and decreased glycolysis, respectively, in NRVMs under hypoxia treated with 25 nM siUCP2 for 48 hours compared with scrambled siRNA controls (*n* = 12 replicates/condition/3 independent experiments). Data for OCR/ECAR were normalized to cell number. (**F**–**H**) Further validation shows increased citrate synthase activity (**F**) and mitochondrial DNA content (**G**), as well as decreased mRNA levels of glycolytic enzymes (**H**) after UCP2 knockdown. (*n* = 3/condition/experiment). (**I**) Increased mitochondrial superoxidase production in NRVMs under hypoxia treated with 25 nM siUCP2 for 48 hours compared with scrambled siRNA controls and measured by MitoSOX intensity. Scale bar: 20 μm (*n* = 3). (**J** and **K**) Increased levels of the DNA double-strand breaks marker γH2A.X (**J**) and the oxidative DNA damage marker 8-OHdG (**K**) in NRVMs under hypoxia after UCP2 knockdown. 8-OHdG/γH2A.X, green; cardiac troponin T, red; nuclei, blue. Scale bar: 40 μm (*n* = 3). (**L**) Increased mRNA expression of DNA damage markers ATM and ATR and decreased antioxidant markers SOD1, GPX, and GR in NRVMs under moderate hypoxia after UCP2 knockdown. (*n* = 3). *P < 0.05, **P < 0.01, ***P < 0.001, ****P < 0.0001. Data from **A–E**, **H**, and **L** were analyzed using Kruskal-Wallis test with Dunn’s correction for multiple comparisons; for **F**, **G**, and **I–K**, Mann-Whitney *U* test was applied.

In previous experiments, we showed that UCP2 levels increase in response to low oxygen levels associated with reprogramming CM metabolism, reduction of oxidative DNA damage, and enhance cell cycle activity. To assess whether UCP2 is required in these processes, we knocked down UCP2 in NRVMs under hypoxia using siRNAs (Supplemental Figure 1C). As expected, the enhancement in cell cycle activity induced by moderate hypoxia (5%) was reverted by UCP2 silencing, demonstrated by decreased levels of Ki67, pHH3, and mRNA expression of positive cell cycle regulators compared with NRVMs treated with scrambled control while cultured under moderate hypoxia (Figure 3, A–C). Seahorse analyses revealed enhanced OCR and decreased ECAR levels (Figure 3, D and E) in NRVMs after UCP2 silencing compared with scrambled control under moderate hypoxia. These alterations were also accompanied by enhanced levels of citrate synthase activity (Figure 3F), enhanced levels mitochondrial DNA content (Figure 3G), decreased expression of glycolytic enzymes (Figure 3H), and enhanced mitochondrial superoxide production (Figure 3I). These findings suggest that UCP2 silencing impairs the hypoxia-induced metabolic switch from mitochondrial oxidative phosphorylation to glycolysis. As a result of the enhanced oxidative metabolism, UCP2-silenced NRVMs exhibited higher levels of the DNA damage markers γ-H2A.X (Figure 3J and Supplemental Figure 3) and 8-OHdG (Figure 3K), mRNA expression of ATM and ATR, and decreased expression of antioxidant genes SOD1, GPX, and GR (Figure 3L) compared with NRVMs treated with scrambled siRNA under moderate hypoxia. Finally, increased levels of TUNEL^+^ CM were also detected after knocking down UCP2 in NRVMs under moderate hypoxia (Supplemental Figure 2B). Collectively, these results highlight the essential role for UCP2 in maintaining CM cell cycle activity and metabolic reprogramming in response to moderate hypoxia.

### Loss of UCP2 reduces cardiac function and CM cell cycle activity in adult mice under moderate hypoxia.

**Figure 4:**
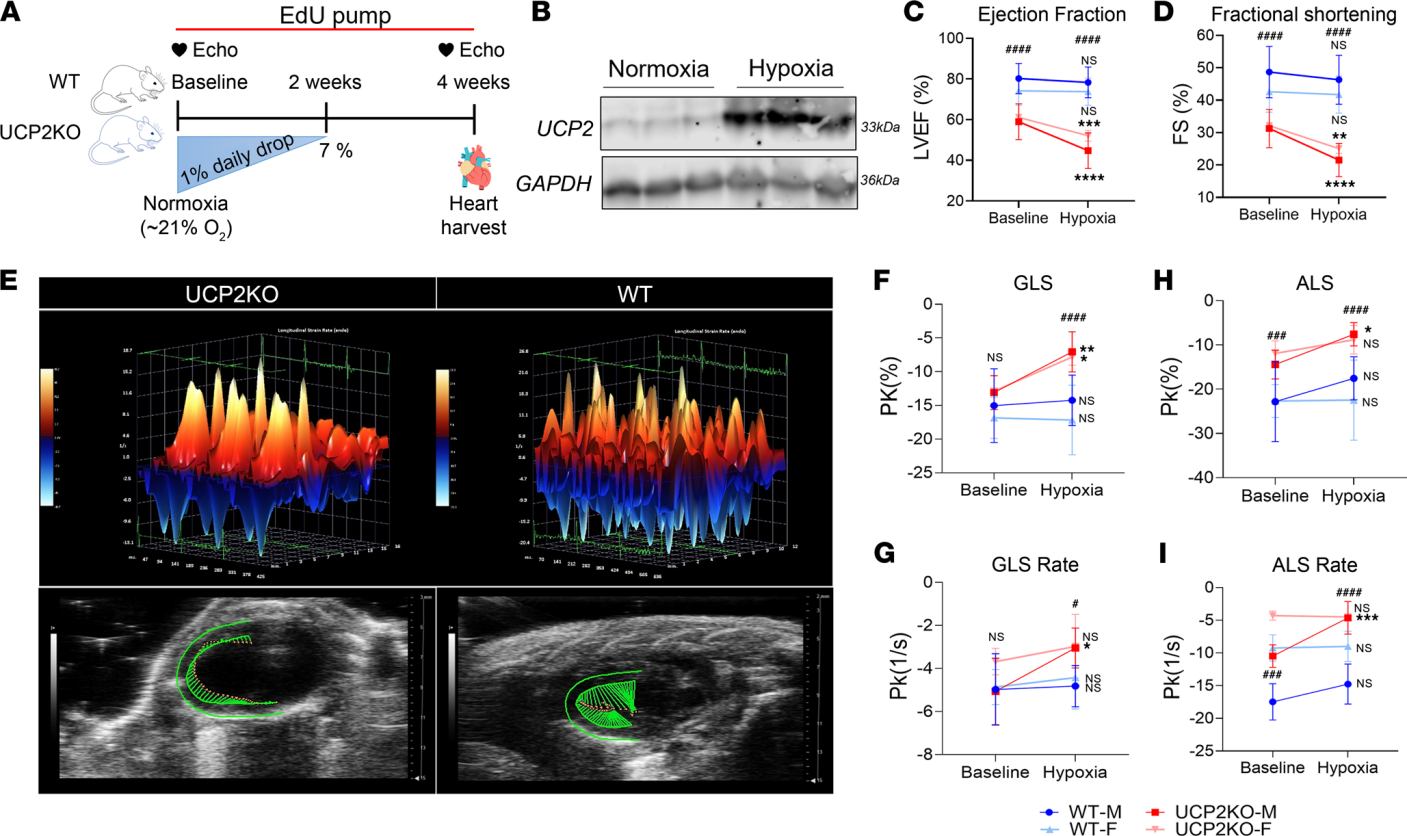
UCP2 ablation decreases cardiac function in adult mice under hypoxia. (**A**) Schematic illustration of the experimental plan shows WT and UCP2KO mice were kept in hypoxic chamber with daily drop of oxygen by 1% until it reached 7%. Mice were kept in 7% hypoxia for an additional 2 weeks, followed by functional and histological assessments. (**B**) Moderate hypoxia treatment increases protein expression of UCP2 in WT mice as measured by immunoblot (*n* = 3 animals/group). LV trace–based analysis of cardiac M-mode images showed decreased cardiac function with lower LV ejection fraction (LVEF) (**C**) and fractional shortening (FS) (**D**) in UCP2KO animals compared with WT mice 4 weeks after hypoxia. Speckle-tracking–based strain imaging. (**E**) 3-dimensional regional wall velocity diagrams show contraction (orange/positive values) or relaxation (blue/negative values) of consecutive cardiac cycles 4 weeks after moderate hypoxia. Vector diagrams show the direction and magnitude of endocardial contraction at mid-systole 4 weeks after moderate hypoxia. (**F**–**I**) Global longitudinal strain (GLS) (**F**), global longitudinal strain rate (GLS rate) (**G**), apical longitudinal strain (ALS) (**H**), and apical longitudinal strain rate (ALS rate) (**I**) in mice lacking UCP2 compared with their WT littermates under hypoxia (*n* = 15–19 male animals and *n* = 8–10 female animals per group). Baseline versus Hypoxia: *P < 0.05, **P < 0.01, ***P < 0.001, ****P < 0.0001; WT versus UCP2KO: ^#^*P* < 0.05, ^###^*P* < 0.001, ^####^*P* < 0.0001. Data from **C**, **D**, and **F**–**I** were analyzed using Kruskal-Wallis test with Dunn’s correction for multiple comparisons.

Our results show that UCP2 is oxygen sensitive and is required for CM metabolic adaptation to low oxygen levels; therefore, we sought whether knocking down UCP2 could impact adult CM cell cycle activity and cardiac function in mice under moderate hypoxia. To answer this question, we utilized a global UCP2-KO mouse line (UCP2KO) and their WT littermates as controls. Both animal groups included males and females, kept in a hypoxia chamber with gradual decrease in oxygen levels (from 21% to 7%) within 2 weeks in accordance with prior literature (8). The mice were kept for an additional 2 weeks at 7% oxygen level followed by various functional and structural analyses (Figure 4A). As shown in Figure 4B, UCP2 protein levels increased in response to hypoxia in the WT mice, confirming the in vitro results in the cell models. Additionally, the increase in UCP2 after hypoxia seems to be more evident in CM than nonmyocytes, suggesting the observed phenotype may in part be due to the loss of UCP2 in CM (Supplemental Figure 4, A and B). Interestingly, mice lacking UCP2 have significantly decreased ability to survive in a hypoxic environment compared with their WT littermates (Supplemental Figure 4C). Further echocardiography analysis revealed an overall decrease in systolic function as measured by analysis of ejection fraction (EF; Figure 4C) and fractional shortening (FS; Figure 4D and Supplemental Figure 4D) in both males and females from the UCP2KO group when compared with the WT 4 weeks after moderate hypoxia, while no difference was observed for the WT group. Although UCP2KO mice have reduced basal cardiac function, our data show that the drop in EF and FS is significantly more in the UCP2KO group than the WT after hypoxia administration. Heart rate (Supplemental Figure 4E) did not change between both groups of animals. Additionally, speckle-tracking–based strain analysis on echocardiography B-mode loops was performed, and Figure 4E shows 3-dimensional regional wall velocity diagrams and vector diagrams for both groups, respectively, 4 weeks after moderate hypoxia treatment. Analysis of global strain (Figure 4F) and rate (Figure 4G) together with apical longitudinal strain (Figure 4H) and rate (Figure 4I) showed significant deterioration of systolic function in UCP2KO animals compared with WT animals 4 weeks after moderate hypoxia treatment.

**Figure 5:**
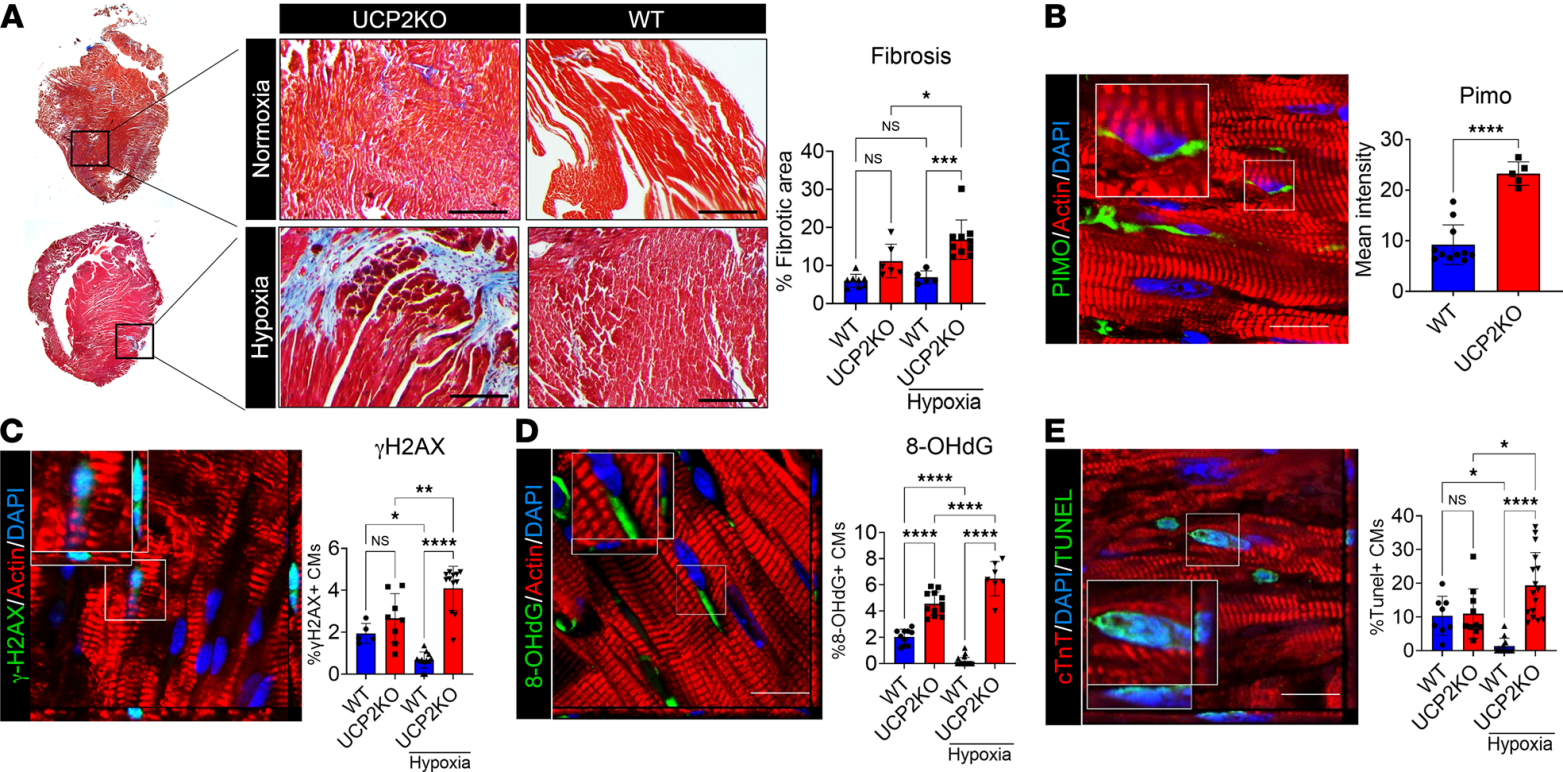
Lack of UCP2 enhances cardiac fibrosis and oxidative DNA damage in adult mice under hypoxia. (**A**) Increased fibrotic area in mice lacking UCP2 under hypoxia compared with their WT littermates, as measured by Masson’s trichome staining 4 weeks after moderate hypoxia. Healthy myocardium, red; fibrotic scar tissue, blue (*n* = 10 animals per group). (**B**) Pimonidazole (PIMO) immunostaining showing increased levels of hypoxic cardiomyocytes in UCP2KO mice under hypoxia (*n* = 10);.(**C** and **D**) Increased levels of the DNA double-strand breaks marker γH2A.X (**C**) and the oxidative DNA damage marker 8-OHdG (**D**) in UCP2KO mice 4 weeks after moderate hypoxia compared with their WT littermates. 8-OHdG and γH2A.X, green; α sarcomeric actin, red; nuclei, blue. Images taken in *Z* stack. Scale bar: 40 μm. (*n* = 10). (**E**) TUNEL staining showing increased apoptotic cardiomyocytes in mice lacking UCP2 under hypoxia compared with WT controls. TUNEL, green; α sarcomeric actin, red; nuclei, blue. Images taken in *Z* stack. Scale bar: 40 μm (*n* = 10). *P < 0.05, **P < 0.01, ***P < 0.001, ****P < 0.0001; WT versus UCP2KO. Data from **A**–**E** were analyzed using Kruskal-Wallis test with Dunn’s correction for multiple comparisons.

Next, hearts were isolated from both groups of male mice, followed by IHC analyses. Masson’s trichome staining revealed an increased interstitial fibrotic area in UCP2KO mice when compared with the WT group in both baseline and 4 weeks after hypoxia (Figure 5A). To analyze changes in the number of hypoxic CM in the heart, WT and UCPKO animals were injected with hypoxic probe pimonidazole (PIMO) prior to harvest of the heart 4 weeks after moderate hypoxia treatment. Results show increased PIMO^+^ hypoxic CM in the UCP2KO animals compared with WT hearts 4 weeks after moderate hypoxia treatment (Figure 5B). Finally, whether hypoxia alters DNA damage and apoptosis in mice was tested. Increased expression of γ-H2A.X (Figure 5C) and 8-OHdG (Figure 5D) was observed in UCP2KO mice compared with WT animals together with increased TUNEL^+^ apoptotic CM 4 weeks after moderate hypoxia (Figure 5E).

**Figure 6:**
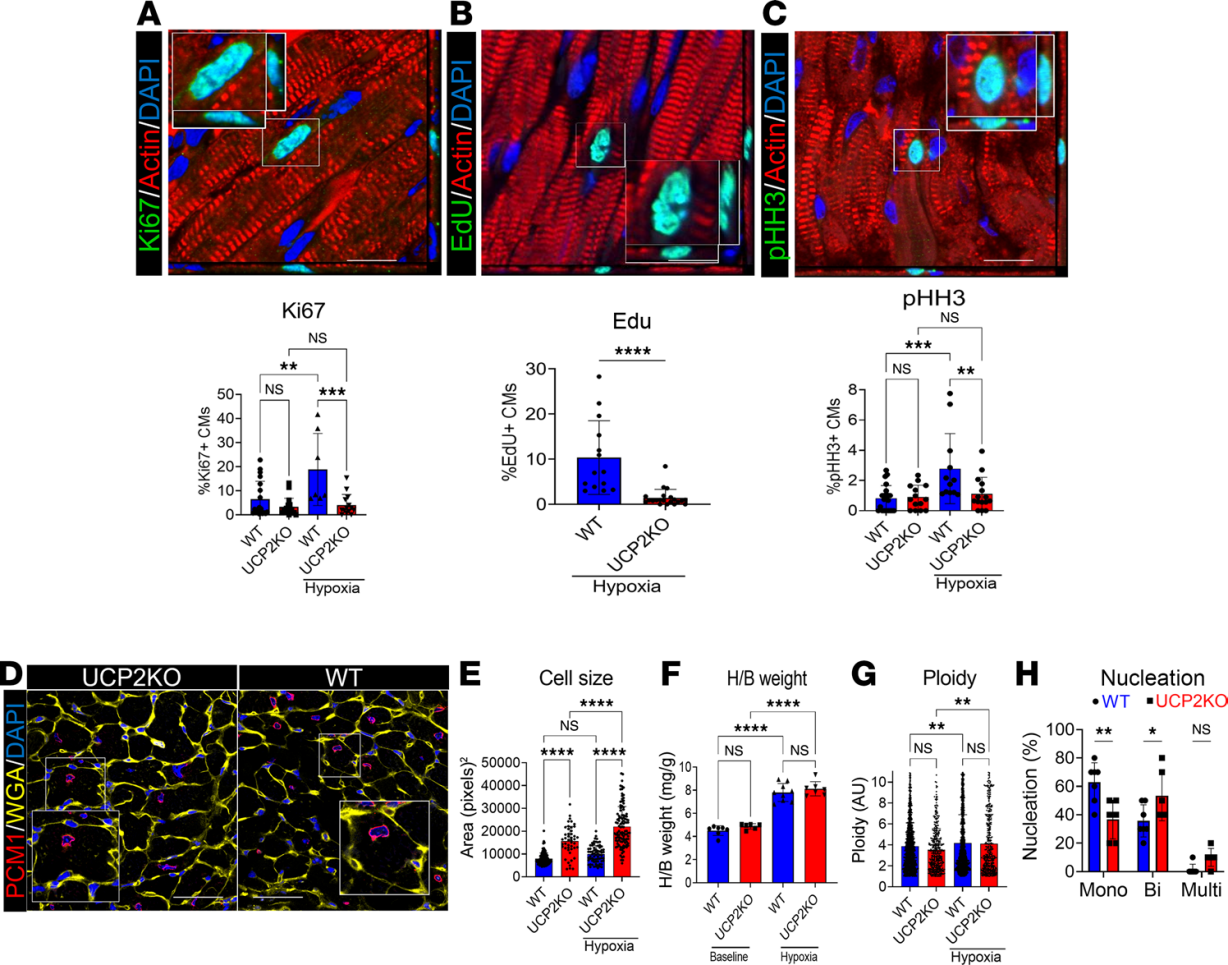
Loss of UCP2 reduce adult CM cell cycle activity in response to hypoxia. (**A**–**C**) Cell cycle activity is significantly reduced in mice lacking UCP2 under hypoxia compared with their WT littermates, as evidenced by decreased levels of Ki67 (**A**), EdU incorporation (**B**), and pHH3 (**C**) 4 weeks after moderate hypoxia. Ki67, EdU, pHH3, green; α sarcomeric actin, red; nuclei, blue. Images taken in *Z* stack. Scale bar: 40 μm (*n* = 10). (**D**) Representative immunostaining image for wheat germ agglutinin (WGA). WGA, yellow; PCM1, red; nuclei, blue. Scale bar: 40 μm. (**E**) Cell size quantification from WGA images showing increased cardiomyocyte size in mice lacking UCP2 (*n* = 10). (**F**) Heart/body weight ratio is increased in UCP2KO mice under hypoxia compared to their littermates (*n* = 10 animals per group). (**G**) Quantification of ploidy levels in PCM1^+^ CM nuclei in WGA-stained images. (**H**) Quantification of the number of nuclei per cardiomyocytes in WGA-stained images showing decreased mononucleated and increased bi- and multinucleated cardiomyocytes in mice lacking UCP2 under hypoxia (*n* = 6). *P < 0.05, **P < 0.01, ***P < 0.001, ****P < 0.0001. Data from **A**–**C**, **F**, and **H** were analyzed using Kruskal-Wallis test with Dunn’s correction for multiple comparisons; for **E** and **G**, 1-way ANOVA with Bonferroni post hoc test for multiple comparisons was applied.

In agreement with our in vitro data, moderate hypoxia treatment enhanced CM cell cycle activity in WT mice as assessed by Ki67 (Figure 6A), EdU (Figure 6B), and pHH3 (Figure 6C) labeling together with increased mRNA expression of cell cycle markers (Supplemental Figure 5A) 4 weeks after moderate hypoxia treatment. However, the same effect was not observed in UCP2KO mice, suggesting that UCP2 is necessary for hypoxia-induced enhancement in CM cell cycle activity. Hypoxia treatment also induced an increase in CM size (Figure 6, D and E) and heart/body weight ratio (Figure 6F), together with increased markers of hypertrophy (Supplemental Figure 5B), in the UCP2KO group when compared with the WT mice. Of note, the observed increase in heart/body weight in WT mice might be related to increased CM proliferation under hypoxia (Figure 6, A–C). No significant difference in ploidy (Figure 6G) and nucleation levels (Figure 6H) were observed. Additionally, metabolic markers of glycolysis (Supplemental Figure 5C) were significantly upregulated in WT mice together with reduced markers of DNA damage and increased antioxidant signaling (Supplemental Figure 5D) 4 weeks after moderate hypoxia.

### UCP2 regulation of CM cell cycle is associated with alterations in acetyl-CoA levels and histone acetylation in CM under moderate hypoxia.

**Figure 7:**
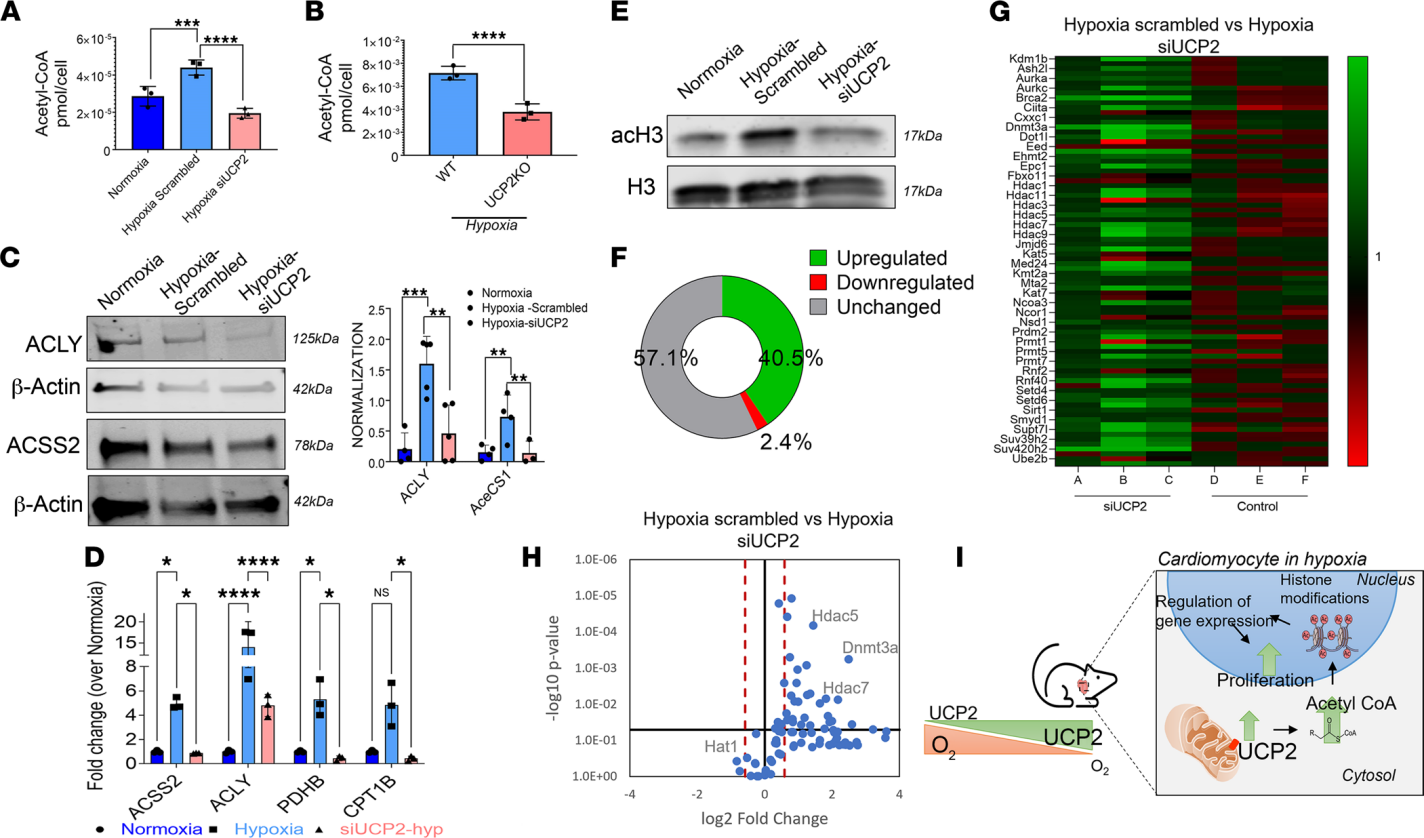
Loss of UCP2 alters acetyl-CoA levels and histone marks in cardiomyocytes. (**A** and **B**) Acetyl-CoA levels are significantly increased in NRVMs (**A**) and adult cardiomyocytes from WT mice (**B**) under moderate hypoxia and decreased after loss of UCP2 as measured by mass spectrometry–based quantification (*n* = 3 per group). (**C** and **D**) Immunoblot and qPCR validations showing increased levels of enzymes and genes involved in acetyl-CoA signaling and reduced after UCP2 silencing in NRVMs under moderate hypoxia (*n* = 3). (**E**) Immunoblot validation showing histone 3 is acetylated in hypoxia and deacetylated after UCP2 silencing in NRVMs. (**F**) Chart resulting from a qPCR array for 84 chromatin remodeling enzymes indicating the percentage of genes upregulated, downregulated, and unchanged after siRNA-mediated silencing of UCP2 in NRVM after 48 hours of moderate hypoxia (*n* = 3). (**G**) Heatmap of the 84 chromatin remodeling enzymes after siRNA-mediated silencing of UCP2 in NRVM under hypoxia. Upregulated, green; downregulated, red (*n* = 3). (**H**) Volcano plot highlighting upregulation of histone deacetylation enzymes and downregulation of a histone acetylation enzyme in the qPCR array after UCP2 silencing. Upregulated < fold change 1.5, downregulated > fold change 1.5 (*n* = 3). (**I**) Schematic illustration summarizing the findings of this study. *P < 0.05, **P < 0.01, ***P < 0.001, ****P < 0.0001. Data from **A**, **C**, and **D** were analyzed using Kruskal-Wallis test with Dunn’s correction for multiple comparisons; for **B**, Mann-Whitney *U* test was applied.

Previous studies show that UCP2 regulates metabolite transport from the mitochondria regulating acetyl-CoA levels (15). Thus, we hypothesized that UCP2 regulates CM cell cycle activity under moderate hypoxia via promotion of the cytosolic generation of acetyl-CoA, which leads to histone acetylation promoting cell cycle gene expression. Indeed, we found acetyl-CoA levels to be increased both in NRVMs after moderate hypoxia (Figure 7A) and adult CM isolated from WT mice (Figure 7B) 4 weeks after moderate hypoxia treatment. However, acetyl-CoA levels were decreased when UCP2 was knocked down both in vitro and in vivo. Next, ATP-citrate lyase (ACLY) and cytoplasmic acetyl-CoA synthetase (ACSS2), which regulates cytosolic generation of acetyl-CoA, were measured by immunoblot analysis and showed a significant increase after moderate hypoxia that was reduced in response to siUCP2 (siRNAUCP2) treatment (Figure 7C) together with increased mRNA expression of the cytosolic acetyl-CoA–generating signaling pathway in both NRVMs (Figure 7D) and adult CM isolated from WT animals 4 weeks after moderate hypoxia (Supplemental Figure 5E) in comparison with UCP2 ablation. To test whether UCP2-induced enhancement in acetyl-CoA could result in histone acetylation, we quantified the amount of acetylated histone 3 (acH3) by Western blot. Interestingly, acH3 was upregulated in NRVMs under moderate hypoxia and decreased in response to siUCP2 (Figure 7E). To further gain molecular insights, we performed a pathway-focused quantitative PCR (qPCR) array for 84 chromatin remodeling enzymes in NRMVs cultured under hypoxia with or without siUCP2. Global profiling showed that UCP2 knockdown led to 40.5% of genes being upregulated and 2.4% being downregulated, while 57.1% remained unchanged in comparison with NRVMs treated with scrambled siRNA under moderate hypoxia (Figure 7, F and G). Interestingly, knocking down of UCP2 under hypoxia significantly upregulated 34 genes, including different histone deacetylases (Hdac1, Hdac5, Hdac6, and Hdac7), and downregulated 2 genes, including histone acetyltransferase 1 (Hat1) (Figure 7H); this suggests that UCP2 levels can influence the balance of histone acetylation/deacetylation, potentially impacting gene expression that regulates CM cell cycle activity under moderate hypoxia.

## Discussion

Our findings here identify a potentially novel role for mitochondrial UCP2 in the heart, as UCP2 may be able to sense changes in oxygen tension, adapting CM cell cycle activity accordingly (Figure 7I). UCP2-mediated changes in CM cell cycle are associated with increased acetyl-CoA cytosolic generation and histone acetylation that possibly regulates expression of cell cycle markers in CM under treatment with moderate hypoxia. Together, these findings link cellular metabolism to oxygen tension in the heart and to regulation of CM cell cycle activity that eventually may lead to the development of novel therapies targeting CM replenishment in the heart after injury.

Transition from the embryonic to postnatal environment is marked by a rapid increase in oxygen tension associated with CM maturation and cell cycle arrest parallel to a shift in energy metabolism toward oxidative phosphorylation (7). Whether there are common metabolic sensors of oxygen tension in the heart regulating CM cell cycle dynamics remains unknown. The premise of the current study is based upon our recent work on UCP2 showing elevated levels in the neonatal heart that promotes glycolytic metabolism and cardiac cell proliferation (17). Here, we utilized in vitro and in vivo models to assess the responsiveness of UCP2 to oxygen levels and its subsequent effect on CM metabolism and cell cycle activity. These results are consistent with our previous work (17) and other studies where UCP2 was found to be enriched during heart development (18, 19) and to be downregulated or absent during adulthood when the heart is highly oxygenated (18). Hypoxia-induced increase in UCP2 has also been reported in cancer cells (20), and its expression in proximal tubular cells promotes the stabilization of HIF-1α through regulation of mitochondrial respiration and oxygen content (21), suggesting that UCP2 may not only sense changes in oxygen levels, but also could trigger adaptative responses. Since UCP2 is a known regulator of glucose metabolism and is upregulated by hypoxia, we sought to investigate the contribution of UCP2 for CM metabolic adaptation to hypoxia (14, 22). As expected, hypoxia-induced UCP2 upregulation promoted a shift toward a more glycolytic metabolism and reduction of oxidative damage. This is consistent with the concept that UCP2 can not only activate proton leak, but can also transport C4 metabolites out of the mitochondria, thus limiting mitochondrial oxidation and reducing ROS production (15, 23). Interestingly, knocking down UCP2 completely reverted hypoxia-induced upregulation of glycolysis with concurrent enhancement in OxPhos and oxidative damage, thus suggesting that UCP2 is essential for metabolic adaptation of CM to hypoxic conditions. This notion is further supported by our in vivo model, where mice lacking UCP2 presented cardiac maladaptation to hypoxia, leading to higher oxidative damage, fibrosis, and worse cardiac function when compared with their WT littermates.

Previous studies have demonstrated that changes in oxygen levels and metabolism are important regulators of CM cell cycle activity (6–8, 11, 13, 24). More specifically, hypoxia exposure has been shown to extend the proliferative window of postnatal CM (7) and induce adult CM proliferation and functional recovery following myocardial infarction. Recently, Ye and colleagues showed that hypoxia induces CM proliferation in the human heart in a population of patients with cyanotic heart diseases (13). Authors categorized patients into 3 main groups based on SaO_2_ levels — mild hypoxia > 85%, moderate hypoxia 75%–85%, and severe hypoxia < 75% — and show that CM proliferation was significantly higher in the moderate hypoxia group. Similarly, moderate hypoxia treatment (3% oxygen) has been shown recently to promote neonatal CM proliferation together with the suggestion that exposure to intermittent hypoxia could be a nonpharmacological therapy to treat cardiovascular disease (25). In concordance, our results show here that moderate hypoxia (5% oxygen) enhances CM cell cycle activity and is dependent on UCP2 upregulation. An increase in UCP2 levels seems to be important for CM adaptation to hypoxic environments, allowing CM to enhance cell cycle activity by reducing mitochondrial-dependent oxidative stress and subsequent oxidative DNA damage, a major contributor to CM cell cycle arrest in accordance with a similar study showing the role of hypoxia in CM proliferation (7). Using both in vitro and in vivo models, we showed that enhanced cell cycle activity is dependent on UCP2, as knocking down UCP2 in hypoxic conditions decreases the expression of cell cycle markers, together with a shift to OxPhos and subsequently enhancing oxidative damage. This is in agreement with previous findings demonstrating that UCP2 is merely found in highly proliferating cells that have a glycolytic and anabolic metabolism, such as cancer and stem cells (19, 26–28), that can shut off mitochondrial respiration by upregulating UCP2, thereby inducing the Warburg effect and enhancing glucose metabolism and lactate production, even in the presence of oxygen (29). Indeed, generating energy preferentially via glycolysis may be a more efficient way to generate metabolic intermediates and nucleotides for further proliferation and to drive angiogenesis in the hypoxic environment.

One of the main findings of the study was the ability of UCP2 to regulate acetyl-CoA levels and histone acetylation, which could potentially affect CM cell cycle activity. Studies show that acetyl-CoA is a central metabolite generated from glucose, amino acid, and fatty-acid catabolism (30). Acetyl-CoA promotes synthesis of citrate that is transported out of the mitochondria by mitochondrial citrate transporter and subsequent cleavage by the enzyme ACLY is well documented and leads to acetylation of key molecules, including histone proteins, in various cell types (31, 32). Growing evidence suggests on-site generation of acetyl-CoA in the nucleus for gene regulation, DNA repair, and mitosis (30). In the cardiac context, distinct epigenetic regulating signaling pathways are known to be altered in fetal, postnatal, and disease CM (33, 34). Our recent study identifies age-dependent changes in acetyl-CoA signaling to histone acetylation in cardiac cells (35). Nevertheless, the precise role of metabolic signaling pathways and histone acetylation for regulation of CM cell cycle in the heart under hypoxia is largely undetermined. In concordance with the above-mentioned role of acetyl-CoA in histone regulation, our data identify that mitochondrial UCP2 alters cytosolic acetyl-CoA levels and histone acetylation, potentially affecting CM cell cycle gene expression in response to moderate hypoxia treatment. We acknowledge that these findings are preliminary, and further validation and deep investigation are still required in order to provide a clear mechanistic link between the loss of UCP2 and changes in histone marks. Although the increase in UCP2 observed in mice under hypoxia is more pronounced in CM than nonmyocytes, we cannot rule out the possibility of the phenotype of the UCP2KO mice being tied to a global loss of UCP2. Lastly, the female mice utilized in the study were only analyzed for a subset of the data, and sex-based differences in the effects of the UPC2 on the cardiac response were not fully explored.

In conclusion, these results provide evidence that UCP2 senses changes in oxygen levels and its upregulation is essential for the metabolic adaptation to hypoxic environments and subsequent enhancement of CM cell cycle activity. Mechanistically, UCP2 promotes cytosolic generation of acetyl-CoA that orchestrates modifications in histone acetylation

## Methods

### Cell Isolation and culture.

NRVMs were isolated from 1- to 2-day-old rat pups as previously described (36) and cultured in F-10 medium (Thermo Fisher Scientific) supplemented with 10% FBS. Adult feline ventricular myocytes were isolated as described previously (37) and cultured in M199 medium supplemented with penicillin, streptomycin, and gentamicin. Hypoxic conditions were introduced gradually beginning at 10% oxygen from 24 to 48 hours and 5% oxygen from 48 to 72 hours.

### Transfection of CM with siRNA.

NRVMs were grown in F10 media without antibiotics and transfected with 25 nM of small interfering RNA (siRNA) for either UCP2 or scrambled control (Horizon Discovery) for 48 hours using DharmaFECT according to the protocol described by the manufacturer.

### qPCR.

Total RNA was isolated from the cells using RNeasy Mini Kit (Qiagen) and reverse transcribed using High-Capacity cDNA Reverse Transcription Kit (Applied Biosystems). qPCR was performed in triplicate using SYBR Green (Bio-Rad) according to the manufacturer’s instructions. Ct levels were normalized to GAPDH and 18 seconds for rat and mouse samples, respectively. Reactions were performed in Bio-Rad CFX96, and were analyzed using the comparative Ct method (2^–ΔΔCt^) Primer information is listed in the Supplemental Table 1.

### Immunoblots.

Immunoblot analysis was performed as previously described (17, 35, 36), with additional detail in Supplemental Methods, including a list of antibodies in Supplemental Table 2.

### Immunostaining and histology.

Immunocytochemistry, IHC, EdU, and TUNEL assays were performed as previously described (17, 36), with additional detail in Supplemental Methods, including a list of antibodies in Supplemental Table 2. Fibrotic area was assessed by Masson’s trichrome staining (Sigma-Aldrich) following protocol described by the manufacturer, and the images were analyzed using ImageJ software (NIH).

### Citrate synthase activity.

Citrate synthase activity was determined spectrophotometrically as directed by the Citrate Synthase Activity Colorimetric Assay Kit (Biovision) with additional detail in Supplemental Methods.

### Mitochondrial DNA.

Mitochondrial DNA copy number was measured as previously described (38), with additional detail in Supplemental Methods.

### ΔΨm.

The ΔΨm was measured as previously described (17) by feeding living cells with 50 nM TMRM (Invitrogen) for 30 minutes at 37°C. Live nuclear dye (Invitrogen) was added to cells 10 minutes prior to scanning on the microscope. Images were taken on Leica SP8 Confocal Microscope. TMRM intensity quantifications were performed on ImageJ.

### Mitochondrial superoxide measurement.

Mitochondrial superoxide was measured by feeding living cells with HBBS supplemented with 2% BSA, 0.06% pluronic acid, 20 μm sulfinpyrazone, and 5 μM MitoSOX (Thermo Fisher Scientific) for 30 minutes at 37°C following live cell imaging at 580 nm. Images were analyzed using ImageJ software and reported as mean intensity.

### Seahorse assays.

A Seahorse Bioscience XF96 Extracellular Flux Analyzer was utilized to measure OCR and ECAR in NRVMs using the Mito Stress and Glycolysis Stress kits (Agilent Technologies), respectively, following the protocol described by the manufacturer. All calculations for assessment of OCR/ECAR were reported as mean ± SD (pmol O_2_/[min mg]).

### Animal studies.

Mice used in this study were obtained from The Jackson Laboratory. Eight- to 12-week-old C57BL/6 male and female mice (WT) and B6.129S4-Ucp2^tm1Lowl^/J (UCP2KOt; stock no. 005934, provided by Tamas Horvath, Yale School of medicine, New Haven, Connecticut, USA) were introduced to hypoxic conditions gradually, beginning at 21% O_2_ and ending at 7% O_2_ after 2 weeks with a 1% daily drop according to a previously described protocol (8). Animals were anesthetized using a mixture of 1.5% isoflurane and oxygen (1 L/min) through inhalation for experimental procedures. All animals were implanted s.c. with osmotic pumps filled with EdU for 4 weeks as previously described (36).

### Echocardiography and hemodynamic assessment.

Transthoracic 2-dimensional B- and M-mode echocardiography was performed at baseline and after 4 weeks of hypoxia treatment using the Vevo2100 (VisualSonics) as described previously (36, 39, 40). Left ventricle (LV) tracing and speckle-tracking–based strain analyses were analyzed using the Vevo Strain Software (Vevo LAB 1.7.1) to determine cardiac function. Additional details in Supplemental Methods.

### Acetyl-CoA measurement.

Acetyl-CoA was quantified in NRVMs under normoxia and hypoxia 48 hours after transfection with siUCP2 (*n* = 3 independent experiments) and in WT and UCP2KO mice after hypoxia treatment (*n* = 10 animals per group) by stable isotope dilution liquid chromatography–high resolution mass spectrometry, as previously described (41, 42); additional details are available in Supplemental Methods. Data were integrated using Tracefinder v4.1 (Thermo Fisher Scientific) software, and additional statistical analysis was conducted by Prism v7.05 (GraphPad). Acetyl-CoA values were normalized to cell number and reported as pmol/1 × 10^5^ cells.

### Statistics.

Statistical analysis is performed using unpaired 2-tailed Student’s *t* test for data comparing 2 groups and 1-way or 2-way ANOVA with Kruskal wallis test with Dunn’s correction or Bonferroni post hoc test for comparing more than 2 groups for data exhibiting normal distribution. For data that do not exhibit normal distribution, Mann-Whitney *U* test was used. All data sets were assessed for normality using Shapiro-Wilk test for normality. *P* < 0.05 is considered statistically significant. Data represent mean ± SD. Statistical analysis is performed using Graph Pad prism v 9.0 software.

### Study approval.

All procedures and animal care protocols were approved by Temple University IACUC in accordance with the *Guide for the Care and Use of Laboratory Animals* (National Academies Press, 2011).

## Author contributions

VOCR, CZ, JK, DH, AE, NK, JJ, MB, LK, and HP performed and analyzed experiments. VOCR, CZ, NK, NS, and MK performed data analysis and interpretation. VOCR, SM, and MK, wrote the manuscript with contributions from SRH. MK contributed conception, design, and financial support.

## Supplementary Material

Supplemental data

## Version 1 06/30/2022

In-Press Preview

## Version 2 08/08/2022

Electronic publication
