# Supplementary material for: UCP2 modulates cardiomyocyte cell cycle activity, acetyl-CoA, and histone acetylation in response to moderate hypoxia
Source: JCI Insight. 2022 Aug 8;7(15):e155475. doi: 10.1172/jci.insight.155475 (PMC9462500; doi:10.1172/jci.insight.155475)
Supplement: Supplemental data [file jciinsight-7-155475-s190.pdf]

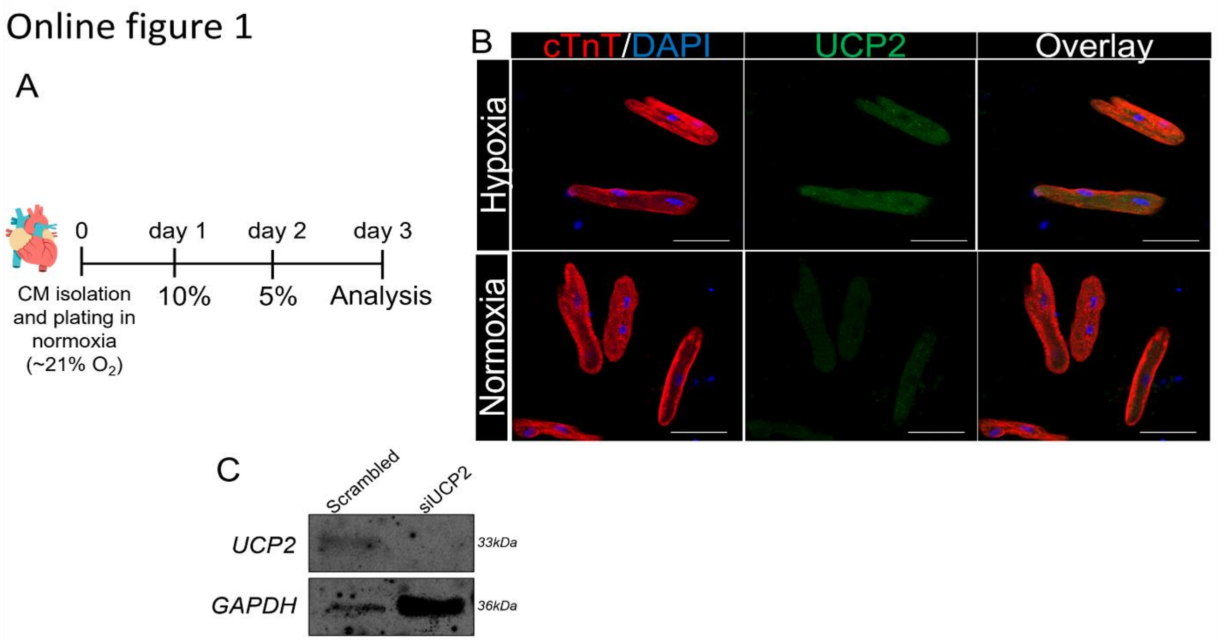

Online Figure 1. A) Schematic illustration of the experimental plan for neonatal rat ventricular myocytes (NRVMs) and adult feline myocytes (AFMs) for treatment under various oxygen concentrations followed cellular and molecular analyses. B) UCP2 is upregulated in AFMs under hypoxia as measured by immunostaining. UCP2 (green), cardiac troponin T (red), nuclei (blue), Scale bar = 20μm. (n=3). C) Immunoblot validation of UCP2 knockdown in NRVMs along with quantitation. (n=3).

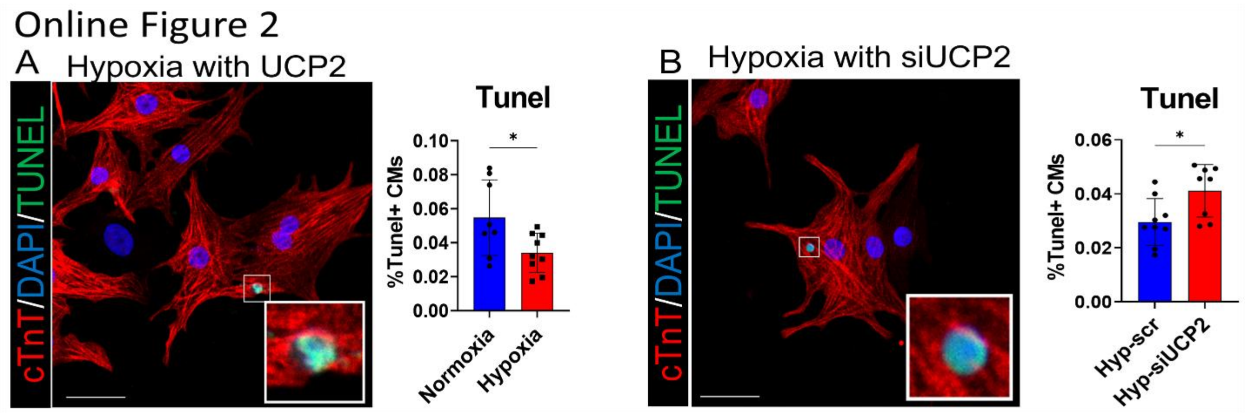

Online Figure 2. A-B) NRVM apoptosis decreases under moderate hypoxia (5% oxygen) (A) and the effect is reversed by silencing UCP2 (B) as measured by TUNEL immunostaining. TUNEL (green), nuclei (blue). Scale bar = 40μm. (n=3). Hypoxia-scrambled vs Hypoxia-siUCP2 *p<0.05, **p<0.01, ***p<0.001, ****p<0.0001. A-B Mann-Whitney test was applied

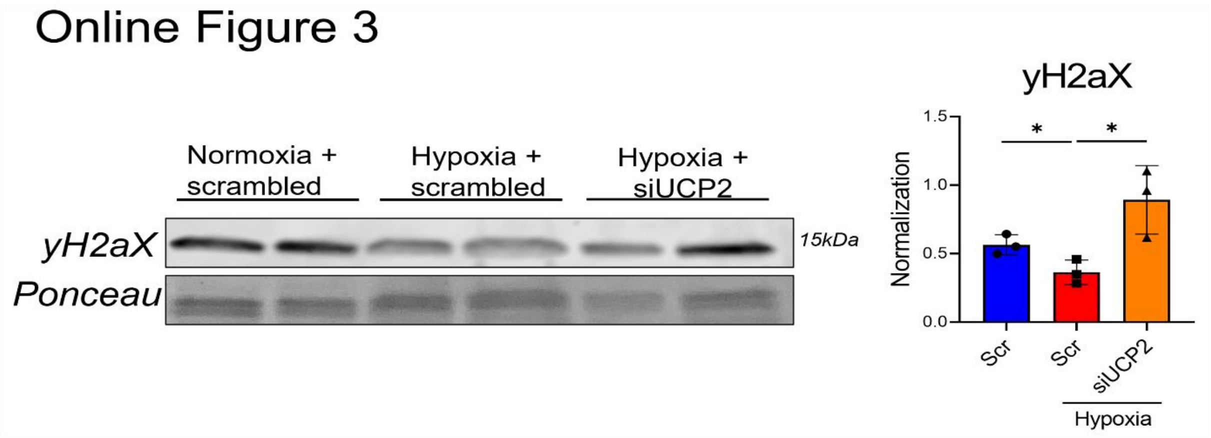

Online Figure 3. Western blot showing yH2AX is increased in NRVMs in hypoxia treated with siUCP2 for 48h.

Online Figure 4

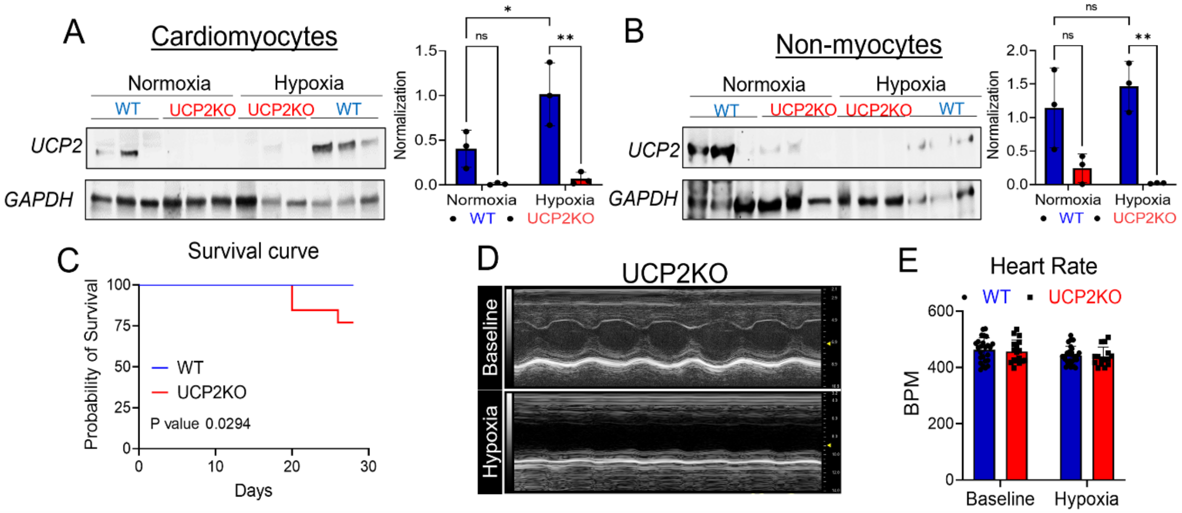

Online Figure 4. A-B) Western blot showing UCP2 protein expression in isolated CMs and non-CMs from WT and UCP2KO mice under normoxia and hypoxia. C) Survival curve showing UCP2KO mice have decreased survival under hypoxic condition. D) Representative M-mode echocardiography image of UCP2KO mice showing reduced cardiac function after hypoxia treatment; E) Heart rate did not differ in both groups (H). n=15-19 male animals per group. Baseline vs Hypoxia *p<0.05. WT vs UCP2KO #p<0.05, ####p<0.0001. Data from E-H was analyzed using Kruskal-Wallis test with Dunn’s correction for multiple comparisons.

Online Figure 5

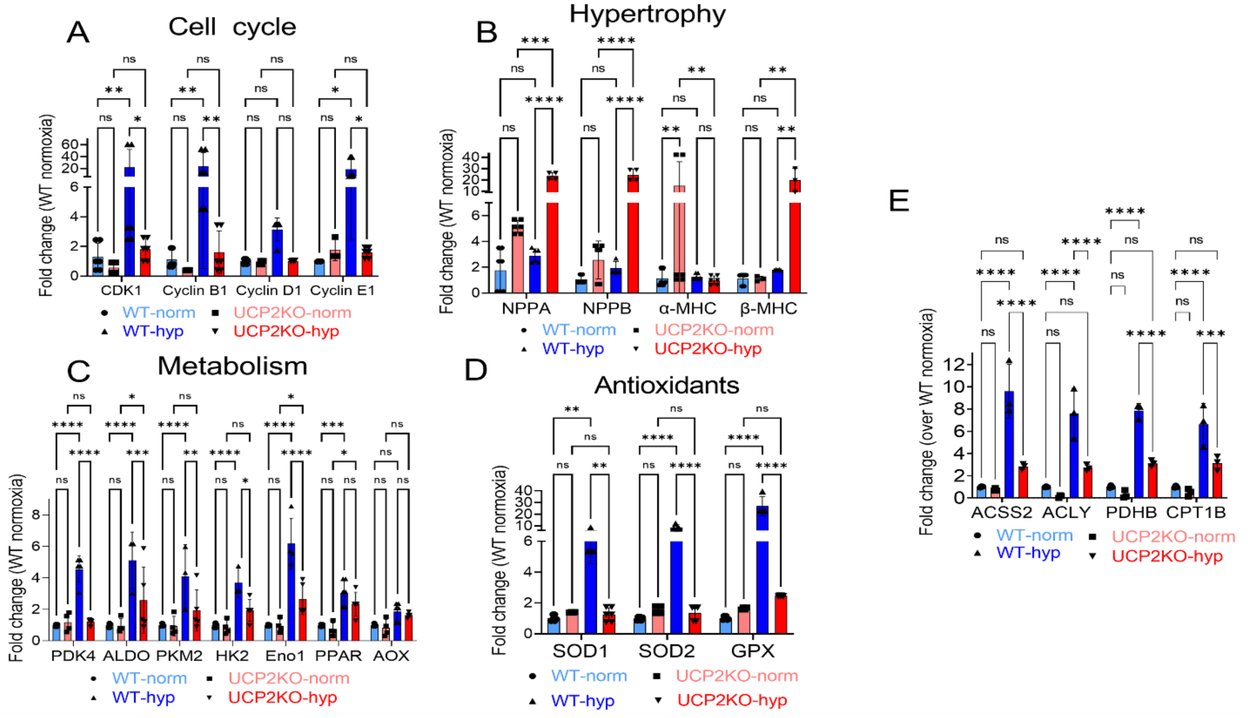

Online Figure 5. mRNA expression of genes involved in cell cycle activity (A), hypertrophic growth (B), metabolism (C), antioxidant response (D), and acetyl-CoA signaling (E). *p<0.05, **p<0.01, ***p<0.001, ****p<0.0001. Data from A-E was analyzed using Kruskal-Wallis test with Dunn's correction for multiple comparisons.

Online Methods

Cell Isolation and culture

Neonatal rat ventricular myocytes (NRVMs) were isolated from 1–2 day old rat pups as previously described(1). NRVMs were plated at a density of 2.5 X 10^5 cells/well in a 2-well chamber slide, 5.0 X 10^5 cells/6-cm dish, and 1.0 X 10^6 cells/10-cm dish. The cells were maintained overnight in F-10 Nutrient Mix (Gibco), supplemented with 10% Horse Serum, Fetal Bovine Serum, and antibiotics (100 units/mL penicillin and 100 mg/mL streptomycin). Adult feline ventricular myocytes were isolated as described previously(2) and cultured in M199 medium supplemented with penicillin, streptomycin and gentamicin. Cells were incubated in a 37°C 5% CO_2 and 20% O_2. Hypoxic conditions were introduced gradually beginning at 10% Oxygen from 24-48 hours and 5% Oxygen from 48-72 hours.

Immunoblots

Immunoblot analysis was performed as previously described(1, 3, 4). In short, protein lysates were loaded onto a 4 – 20% Mini-PROTEAN TGX Gel (Biorad) for electrophoresis. Separated proteins were then transferred onto a nitrocellulose membrane, washed with 1X PBS, and blocked with PBS-based Licor Buffer for 1 hour at 4°C. This was followed by primary antibody incubation overnight. Respective secondary antibody cocktails were added for 90 minutes and fluorescence signal was detected and quantified using Odyssey CLx Imager (Licor) and ImageJ.

Immunostainings and histology

Immunocytochemistry, EdU and TUNEL assays were performed as previously described(1, 5). Immunostaining of NRVMs was performed on cells grown on permanox or glass chamber slides. Cells were fixed by 4% paraformaldehyde (PFA), permeabilized in PBS supplemented by 0.25% Triton-X for 10 min and blocked in PBS supplemented with 10% horse serum for 1 hr. Primary

antibodies diluted were applied overnight at 4°C after blocking in PBS with 10% horse serum. The next day, cells were washed with PBS and incubated for 1 h at room temperature with secondary antibodies diluted in blocking solution and then stained for DAPI (1:10.000) for 10 minutes. Vectashield (Vector Labs, CA, USA) mounting media was used to prepare the slides.

Paraffin heart sections were deparaffinized in xylene and rehydrated through graded alcohols to distilled water. Antigen retrieval was achieved by boiling the slides in 10 mmol/L citrate pH 6.0 for 12–15 min. Slides were washed several times with distilled water and once with TN buffer (100 mmol/L Tris, 150 mmol/L NaCl). Slides were then washed in TN buffer and blocked in TNB buffer (TSATM kit from Perkin-Elmer) at room temperature for at least 1 hr. Primary antibodies were applied overnight at 4°C in TNB buffer. The next day, samples are washed in TN buffer and incubated with secondary antibodies at room temperature in the dark for 1 hr. Slides are washed in TN buffer and coverslipped using Vectashield in the presence of DNA staining. List of primary and secondary antibodies is reported in Table S2.

EdU labelling and apoptotic cells were assessed by using Click-iT EdU Alexa Fluor 488 Imaging Kit (Thermo Fisher Scientific) and Click-iT™ Plus TUNEL Assay (Thermo Fisher Scientific), respectively, as described by the manufacturer. Fibrotic area was assessed by Masson's trichrome staining (Sigma-Aldrich) following protocol described by the manufacturer and the images were analyzed using ImageJ software.

Citrate Synthase Activity

Citrate synthase activity was measured using citrate synthase kit (Biovision) according to manufacturer's protocol. Briefly, NRVMs were grown to confluency followed by lyses using ice-cold CS Assay Buffer. Lysate was kept on ice for 10 minutes then centrifuged at 10,000 RPM for 5 minutes. Once supernatant was collected, samples were added to a 96-well flat bottom plate. Total volume of 50µL was achieved by adding appropriate amount of CS Assay Buffer. Diluted CS Positive Control was added in specific wells and adjusted to total 50µL with CS Assay Buffer.

Standard curve preparation involved diluting GSH Standard and adding to wells. Reaction mix consisting of CS Assay Buffer, CS Developer, and CS Substrate Mix was then added to each well containing either sample, Positive Control, or Standards. Absorbance (OD 412 nm) was measured in kinetic mode at 25°C for 20-40 minutes. Calculation of citrate synthase activity was done using manufacturer's equation.

Mitochondrial DNA

For assessment of Mitochondrial DNA content, cells were centrifuged into a pellet, supernatant was aspirated and DNA lysis buffer (0.5% SDS, 0.1M NaCl, 0.05M Tris (pH 8.0), 3mM EDTA, and ddH20) was added to cells. Proteinase K is added at 100µg/mL to digest any contaminating proteins. Samples were incubated at 60°C overnight and then mixed with 8M Potassium Acetate. Chloroform was added to sample followed by centrifugation for 5 minutes at 9500rpm. Aqueous phase was isolated and 100% EtOH was added and stored in -20°C freezer for 15 minutes. After centrifugation for 5 minutes at 13000 rpm, supernatant was removed, and pellet was washed with 75% EtOH. Samples were centrifuged for 2 minutes at 1300 rpm and supernatant was removed. Once air-dried, samples were resuspended in 100 µL ddH2O. DNA samples were normalized to 100ng/µl. qPCR was performed with primers for β-globin representing nuclear genome and COXII for mitochondrial genome. Mitochondrial genome content was measured after normalizing samples.

Echocardiography

Transthoracic two-dimensional echocardiography was performed at baseline and after 4 weeks of hypoxia treatment using the Vevo2100 (VisualSonics, Toronto, ON, Canada) equipped with a 30-MHz transducer. B and M-mode measurements were recorded for all mice under anesthesia with a mixture of 1.5% isoflurane and oxygen (1 L/min). M-mode tracings were used to measure left ventricular wall thickness and left ventricular inner diameter in systole and diastole. The mean

value of three measurements was determined for each sample. Percentage fractional shortening and percentage ejection fraction are calculated as described previously(1, 5). Speckle-tracking based strain analysis on echocardiography B-mode loops with a frame rate ≥200 frame/second and consisted of 300 frames was also performed for all animal groups. All images were analyzed using the Vevo Strain Software (Vevo LAB 1.7.1) for strain, which evaluates changes in length relative to the initial length (strain = final length [L]/initial length [L0]), calculated either in the radial axis (from the center of the ventricle cavity outward) or longitudinal axis (from the apex to the base). The strain rate (SR), which is the rate of change of this deformation over time (SR = strain/time), was also measured. Global and regional (six segments: basal, mid, and apical anterior; basal, mid, and apical posterior) LV endocardial longitudinal and radial strain (peak strain %), as well as SR were evaluated. Regional LV endocardial longitudinal and radial strain/SR were reported as apical versus mid versus basal segments and as anterior (average of basal, mid, and apical) versus posterior (average of basal, mid, and apical).

Acetyl Co-A measurement

Acetyl-CoA were quantified by stable isotope dilution liquid chromatography-high resolution mass spectrometry, as previously described(6, 7). Cell pellets were spiked with a 13C315N1-internal standard mixture biosynthetically prepared as previously published and sonicated for 12 cycles of 0.5 sec. pulses in 10% (w/v) trichloroacetic acid (Sigma Aldrich) in water. Protein was pelleted by centrifugation at 17,000rcf for 10 min. at 4ºC. The cleared supernatant was purified by solid-phase extraction using Oasis HLB 1cc (30 mg) SPE columns (Waters). Columns were washed with 1 mL methanol, equilibrated with 1 mL water, loaded with sample, desalted with 1 mL water, and eluted with 1 mL methanol containing 25 mM ammonium acetate. The purified extracts were evaporated to dryness under nitrogen and resuspended in 55 µl 5% (w/v) 5-sulfosalicylic acid (SSA) in optima HPLC grade water. Acetyl-CoA was measured by liquid chromatography-high

resolution mass spectrometry. Briefly, 5 µl of sample in 5% SSA were analyzed by injection into an Ultimate 3000 HPLC coupled to a Q Exactive Plus (Thermo Scientific) mass spectrometer in positive ESI mode using the settings described previously. Calibration curves were prepared using commercially available standards from Sigma Aldrich and processed identically as the samples. Data were integrated using Tracefinder v4.1 (Thermo Scientific) software, and additional statistical analysis conducted by Prism v7.05 (GraphPad). Acetyl-CoA values were normalized to cell number and reported as pmol/105 cells.

RT2 Profiler Array

Total RNA was isolated from cultured NRVMs using RNeasy Mini Kit (Qiagen) with DNase I (Qiagen) treatment. Total RNA was then reverse transcribed using the First Strand Synthesis Kit (Qiagen) and subsequently loaded on to Epigenetic Chromatin Modification Enzymes RT^2 profiler array according to manufacturer's instructions (Qiagen). Qiagen's online web analysis tool was utilized to produce comparative heat map, and volcano plot. Fold change was calculated by determining the ratio of mRNA levels to control values using the ΔCt method ($2^{-\Delta\Delta Ct}$). All data were normalized to an average of four housekeeping genes Actb, Gapdh, B2m, Rplp1, and Hprt.

Table S1 – List of primers

Specie	Target	Orientation	Sequence
Rat	ACLY	Forward	GTCCCAAGTCCAAGATCCCTG
		Reverse	CACTGAAGGCTCATCTCGGG
	ACSS2	Forward	CCTTGGAGAAGTGCCGAGAG
		Reverse	AGGAGATCTGGACATCTGGACA
	ALDO1	Forward	CCTTAGTCCTTTCGCCTACCC
		Reverse	CGTTGCCATGGGTCACCTTG
	CDK1	Forward	GGAACAGAGAGGGTCCGTTG
		Reverse	GCACTCCTTCTTCCTCGCTT
	CDK4	Forward	GAGGGGGCCTCTCTAGCTC
		Reverse	TCAGCCACGGGTTCATATCG
	COXII	Forward	GGCTTACAAGACGCCACATC
		Reverse	TCTTGGGCGTCTATTGTGCT
	CPT1b	Forward	CAGTTCACAGGCATAAGGGGT
		Reverse	CACTCCAATCCCACCTCGACC
	Cyclin A2	Forward	GGATGGTAGTTTTGAATCACCCC
		Reverse	GTGATGTCTGGCTGCCTCTT
	Cyclin B1	Forward	TGAACTTCAGTCTGGGTCGC
		Reverse	GGCAAAATGCACCATGTCGT
	Cyclin D2	Forward	GCTCTGTGTGCTACCGACTT
		Reverse	CACATCGGTGTGGGTGATCT
	Cyclin E1	Forward	GACAGCTAGCGCGGTGTAG
		Reverse	TTGGAACTCAGACCCGAAGC
	GAPDH	Forward	GAAGCTCATTTCCTGGTATGACA
		Reverse	TATTGATGGTATTCGAGAGAAGGG
	GPX	Forward	ACTTGAGGGAATTCAGAATCTCTTC
		Reverse	GACTGGTGGTGCTCGGTT
	GR	Forward	GGG CAA AGA AGA TTC CAG GTT
		Reverse	GGA CGG CTT CAT CTT CAG TGA
	PDHB	Forward	ATAGGTCTCACCCTGCCAAAG
		Reverse	CCGAATGACAACACAGAAGCG
	PDK1	Forward	CCGGGCAGCGGCATAGAG
		Reverse	CCTTGCCAGCCTCATACCGAG
	PDK4	Forward	ACA GGA AAC CCA AGC CAC AT
		Reverse	GCA CAC TCA AAG GCA TCT TCG
	PKM2	Forward	CGCCTGGACATTGACTCTG
		Reverse	GAAATTCAGCCGAGCCACATT
	SOD1	Forward	GCTTCTGTCGTCTCCTTGCT
		Reverse	CTCGAAGTGAATGACGCCCT
	UCP2	Forward	GAGACCTCAAAGCACCCTCC
		Reverse	CAATACAGGCTGCTGTCCCA

Mouse	18s	Forward	ACGAGACTCTGGCATGCTAACTAGT
		Reverse	CGCCACTTGTCCCTCTAAGAA
	ALDO1	Forward	CCTTAGTCCTTTCGCCTACCC
		Reverse	CGTTGCCATGGGTCACCTTG
	aMHC	Forward	GGACATTGGTGCCAAGAAGAT
		Reverse	AGGGTCTGCTGGAGAGGTTAT
	bMHC	Forward	TCCTGCTGTTTCCTTACTTGCT
		Reverse	GCTGAGGCTTCCTTTCTCGG
	CDK1	Forward	AAGTGTGGCCAGAAGTCGAG
		Reverse	TCGTCCAGGTTCTTGACGTG
	CPT1	Forward	ACTGAGACTGTGCGTTCCTG
		Reverse	GTGCTTTTCGGAGGCTTTCC
	Cyclin B1	Forward	TCTCGAATCGGGGAACCTCT
		Reverse	GCCATACTGACCTTGGCCTT
	Cyclin D1	Forward	CAAGTGTGACCCGGACTGC
		Reverse	TTGACTCCAGAAGGGCTTCAA
	Cyclin E1	Forward	CAC GGG TGA GGT GCT GAT
		Reverse	AGG ACG CAC AGG TCT AGA AGC
	Eno1	Forward	TCCTTAAGGCTCTCCTCGGT
		Reverse	AGTAGGATCGCTGCAAAGCA
	GPX	Forward	ACTTGAGGGAATTCAGAATCTCTTC
		Reverse	GACTGGTGGTGCTCGGTT
	HK2	Forward	GAT CGT TGG AGC AGACCA CA
		Reverse	TGT ACA AAC ACC CCG AGA CG
	NPPA	Forward	TCGGAGCCTACGAAGATCCA
		Reverse	GTGGCAATGTGACCAAGCTG
	NPPB	Forward	GCCAGTCTCCAGAGCAATTCA
		Reverse	AGCTGTCTCTGGGCCATTTC
	PDK4	Forward	GCT GCT GGA CTT TGG TTC AGA
		Reverse	CGG TCA GGC AGG ATG TCA AT
	PKM2	Forward	TTC GCA TGC AGC ACC TGA TA
		Reverse	TTC AAA CAG CAG ACG GTG GA
	PPAR	Forward	TGCAAACTTGGACTTGAACG
		Reverse	GATCAGCATCCCGTCTTTGT
	SOD1	Forward	AGC ATG GGT TCC ACG TCC
		Reverse	CATGGTTCTTAGAGTGAGGATTAAAAT
	SOD2	Forward	CACCATTTTCTGGACAAACCTG
		Reverse	TTAAACTTCTCAAAAGACCCAAAGTC

Table S2 – List of antibodies

Application	Target	Dilution	Company	Cat#
ICC/Western blot	UCP2	1:50 / 1:500	Santa Cruz	sc-390189
ICC/IHC	Ki67	1:50	abcam	Ab15580
ICC/IHC	pHH3	1:50	abcam	ab47297
ICC/IHC	AurB	1:50	abcam	ab2254
ICC/IHC	8-OHdG	1:50	abcam	ab48508
ICC/IHC	γH2AX	1:50	abcam	ab81299
IHC	Sarcomeric Actin	1:100	Sigma	A2172
ICC/IHC	cTnT	1:100	abcam	ab188877
IHC	PCM1	1:50	Atlas	HPA023374
IHC	WGA	1:100	Invitrogen	W32464
IHC	hypoxy probe-1	1:50	HPI	90132-50-3
Western Blot	ACSS2	1:500		
Western Blot	ACLY	1:500		

1. Borden A, Kurian J, Nickoloff E, Yang Y, Troupes CD, Ibetti J, et al. Transient Introduction of miR-294 in the Heart Promotes Cardiomyocyte Cell Cycle Reentry After Injury. *Circ Res.* 2019;125(1):14-25.
2. Nuss HB, and Houser SR. T-type Ca2+ current is expressed in hypertrophied adult feline left ventricular myocytes. *Circ Res.* 1993;73(4):777-82.
3. Kurian J, Bohl V, Behanan M, Mohsin S, and Khan M. Transcriptional Profiling of Cardiac Cells Links Age-Dependent Changes in Acetyl-CoA Signaling to Chromatin Modifications. *Int J Mol Sci.* 2021;22(13).
4. Kurian J, Yuko AE, Kasatkin N, Rigaud VOC, Busch K, Harlamova D, et al. Uncoupling protein 2-mediated metabolic adaptations define cardiac cell function in the heart during transition from young to old age. *Stem Cells Transl Med.* 2020.
5. Khan M, Nickoloff E, Abramova T, Johnson J, Verma SK, Krishnamurthy P, et al. Embryonic stem cell-derived exosomes promote endogenous repair mechanisms and enhance cardiac function following myocardial infarction. *Circ Res.* 2015;117(1):52-64.
6. Frey AJ, Feldman DR, Trefely S, Worth AJ, Basu SS, and Snyder NW. LC-quadrupole/Orbitrap high-resolution mass spectrometry enables stable isotope-resolved simultaneous quantification and (1)(3)C-isotopic labeling of acyl-coenzyme A thioesters. *Anal Bioanal Chem.* 2016;408(13):3651-8.
7. Snyder NW, Tombline G, Worth AJ, Parry RC, Silvers JA, Gillespie KP, et al. Production of stable isotope-labeled acyl-coenzyme A thioesters by yeast stable isotope labeling by essential nutrients in cell culture. *Anal Biochem.* 2015;474:59-65.
